# Triazole Derivatives Target 14α–Demethylase (LDM) Enzyme in *Candida albicans* Causing Ergosterol Biosynthesis Inhibition

**DOI:** 10.3390/jof8070688

**Published:** 2022-06-29

**Authors:** Irfan A. Rather, Jamal S. M. Sabir, Amer H. Asseri, Mohmmad Younus Wani, Aijaz Ahmad

**Affiliations:** 1 Department of Biological Sciences, Faculty of Science, King Abdulaziz University (KAU), Jeddah 21589, Saudi Arabia; jsabir@kau.edu.sa; 2 Centre of Excellence in Bionanoscience Research, King Abdulaziz University (KAU), Jeddah 21589, Saudi Arabia; 3 Biochemistry Department, Faculty of Science, King Abdulaziz University (KAU), Jeddah 21589, Saudi Arabia; ahasseri@kau.edu.sa; 4 Department of Chemistry, College of Science, University of Jeddah, Jeddah 21589, Saudi Arabia; 5 Clinical Microbiology and Infectious Diseases, Faculty of Health Sciences, School of Pathology, University of the Witwatersrand, Johannesburg 2193, South Africa; 6 Academic Hospital, National Health Laboratory Service, Infection Control Unit, Charlotte Maxeke Johannesburg, Johannesburg 2193, South Africa

**Keywords:** triazole derivatives, *C. albicans*, ergosterol, molecular docking, simulation

## Abstract

*Candida albicans* is the most dominant and prevalent cause of fungal infections in humans. Azoles are considered as first-line drugs for the treatment of these infections. However, their prolonged and insistent use has led to multidrug resistance and treatment failures. To overcome this, modification or derivatization of the azole ring has led to the development of new and effective antifungal molecules. In a previous study, we reported on the development of new triazole-based molecules as potential antifungal agents against *Candida auris*. In this study, the most potent molecules from the previous study were docked and simulated with lanosterol 14-alpha demethylase enzyme. These compounds were further evaluated for in vitro susceptibility testing against *C. albicans*. In silico results revealed favorable structural dynamics of the compounds, implying that the compounds would be able to effectively bind to the target enzyme, which was further manifested by the strong interaction of the test compounds with the amino acid residues of the target enzyme. In vitro studies targeting quantification of ergosterol content revealed that pta1 was the most active compound and inhibited ergosterol production by >90% in both drug-susceptible and resistant *C. albicans* isolates. Furthermore, RT-qPCR results revealed downregulation of *ERG11* gene when *C. albicans* cells were treated with the test compound, which aligns with the decreased ergosterol content. In addition, the active triazole derivatives were also found to be potent inhibitors of biofilm formation. Both in silico and in vitro results indicate that these triazole derivatives have the potential to be taken to the next level of antifungal drug development.

## 1. Introduction

The increasing number of immunocompromised patients has drastically increased fungal infections [1,2]. *Candida* has been reported to be responsible for causing high incidences of mucosal infections and has been graded as second in fungal infections and fifth in nosocomial infections [3,4]. There are 20 *Candida* species capable of causing candidiasis; however, *Candida albicans* (*C. albicans*) appears to be the most common [5,6]. Since *C. albicans* is a eukaryotic organism with a high degree of genetic similarity with its human host, it is challenging to determine unique drug targets specific to fungal cells and lacking in human cells. One such unique target is ergosterol and its biosynthesis pathway. For that reason, targeting the ergosterol biosynthesis pathway has widely been used as an approach to develop new antifungal drugs such as azoles and polyenes [7,8]. However, most of these antifungal drugs belonging to different classes are associated with several side effects such as toxicity. Of note, amphotericin B is nephrotoxic, hepatotoxic, and causes infusion-related reactions and hypokalemia; flucytosine is known to cause anemia and neutropenia. All azoles except fluconazole are known to cause hematopoiesis and inhibit macrophage colony-forming units in the human bone marrow; they are also associated with narrow spectrum and emergence of drug resistance [9,10]. Triazoles have been used to treat *Candida* infections for decades and are considered the building blocks of azole-based antifungals [11]. Fluconazole, the most widely used antifungal drug, is fungistatic and overused, which enormously contributed to developing drug resistance against *Candida* species [12]. With the huge success of different azoles, despite having the same drug target but with different pharmacokinetic properties, they have been introduced to the clinics to treat fungal infections. Being fungistatic with only one drug target, several drug resistance mechanisms have been studied in *C. albicans* against azole drugs, e.g., over-production of ergosterol, biofilms, and efflux pumps amplifies the need for new antifungal drugs. Thus, bringing appropriate structural changes or modifications to the current azole drugs may be an effective strategy for overriding the need to develop new antifungal drugs. This strategy of structurally modifying existing drugs to synthesize new ones with multiple drug targets will help circumvent or obfuscate drug resistance mechanisms in fungal pathogens [13,14].

In our previous study, we reported on the promising antifungal activity of triazole derivatives against multidrug-resistant *C.* *auris* [15]. The results reinforce that these compounds have the potential to induce apoptosis and cell cycle arrest in *C. auris*. In continuation to the previous study and based on the promising results, we further evaluated the antifungal activity of these derivative against different isolates of *C. albicans*. Therefore, in this study, the most active piperidine-based 1,2,3-triazolylacetamide derivatives were docked and simulated with lanosterol 14-alpha demethylase enzyme. Based on the in-silico observations, the piperidine derivatives were further evaluated for in vitro susceptibility testing against *C. albicans*. Furthermore, the effects of these derivatives were tested on ergosterol biosynthesis and the expression of the *ERG11* gene, which is related to the ergosterol biosynthesis pathway.

## 2. Materials and Methods

### 2.1. Candida Strains

In this study, one clinically isolated fluconazole susceptible (*C. albicans* 4175) and one fluconazole resistant (*C. albicans* 5112) were tested in addition to laboratory strain *C. albicans* SC5314. Both the clinical isolates were previously obtained from different clinics in Johannesburg Academic Hospital and were stored at −80 °C in glycerol or maintained on sabouraud dextrose agar (SDA) plates as described elsewhere [5].

### 2.2. Synthesis of Triazole Derivatives

The synthesis of six piperidine-based 1,2,3-triazolylacetamide derivatives (pta1–pta6) has already been reported previously [15] (Scheme S1). The antifungal susceptibility against *C. auris* was also determined. The most potent antifungal compounds (pta1–pta3) were selected for this study to determine their antifungal activity by targeting the ergosterol biosynthesis pathway of *C. albicans*.

### 2.3. In Silico Methodology

#### 2.3.1. Molecular Docking

The three-dimensional X-ray crystal structure of lanosterol 14-alpha demethylase enzyme (LDM) was claimed from the Protein Data Bank database under the PDB accession code of 5V5Z (https://www.rcsb.org/structure/5v5z (accessed on 31 July 2021)). Protein Preparation Wizard implemented in the Schrödinger suite was used to preprocess, minimize, and refine enzyme structure [16]. The enzyme structure was assigned a precise charge and protonation state at pH 7.0 by eliminating crystallographic waters and resolving any missing side chains or hydrogen atoms. This identifies the appropriate ionization state for basic and acidic amino acid residues. An RMSD cut-off value of 0.30 Å was used to minimize energy losses due to the addition of hydrogen atoms to the structure to reduce steric clashes between the residues [17].

The compounds (pta1, pta2, and pta3) used in this study were sketched using MarvinSketch 6.2.1, 2014, ChemAxon (https://www.chemaxon.com (accessed on 31 July 2021)). Fluconazole drug (PubChem ID: 3365) was also used as a positive control, retrieved from the PubChem database (https://pubchem.ncbi.nlm.nih.gov/substance/46505735 (accessed on 31 July 2021)). All these compounds were then processed using LigPrep module implemented in the Schrödinger package (https://www.schrodinger.com/ligprep (accessed on 31 July 2021)). This step detailed the hydrogen atom addition by adjusting realistic bond lengths and angles, accurate chiralities, stereo chemistries, ionization, tautomers, and conformations of ring structures. Partially charged compounds were assigned based on the forcefield generated by OPLS-2005. All the compounds were applied until the RMS value reached 0.001 Å. The ionization state was fixed by using the Epik tool [18].

To identify the best binding scores for the LDM enzyme, Glide docking was performed using Maestro 12.2 [19]. The receptor grid was created using two cubical boxes with a common centroid (X = −36.95 Y = −18.09 Z = 26.72) and using these boxes to arrange the calculations: the larger enclosing box was 25 × 25 × 25 Å, while the smaller binding box was 15 × 15 × 15 Å. Considering the size of the complex and the number of ligands present, the grid box was positioned at the centroid of the ligands to maximize the amount of enzyme structure explored. The docking protocols used were “Extra-Precision” (XP) and “Convolutional”. We used docking and XP-G scores to evaluate the studied compounds, followed by molecular dynamics simulations.

#### 2.3.2. Molecular Dynamics (MD) Simulations

MD simulation is one of the most fundamental approaches to understanding the structural function and dynamics of biomacromolecules [20]. To perform MD simulations on the molecules, GPU accelerated simulation engine PMEMD under AMBER 18 package was used [21]. For assigning the ligands’ atomic partial charges (partial charges of the atoms), the ANTECHAMBER module [22] was used; AMBER 18 forcefield FF14SB [23] was used to define a protein. The complexes were completely solvated within 10 Å box edges with LEaP module of AMBER 18 [22] using TIP3P virtual water boxes [24]. Before the minimization phase, Cl^−^ and Na^+^ counterions were added to neutralize the complexes using the same LEaP module. Using a 500 kcal/mol restraint potential gradient, part-minimization of 1500 steps was further achieved, and a complete minimization of 1000 steps was achieved by eliminating all applied restraints. Afterward, each complex was gradually heated from 0 K to 300 K for 50 ps to maintain its volume and number of atoms. The collision frequency was set at 1 ps on all the complexes’ solutes with 10 kcal/mol potential harmonic restriction. In the following step, equilibration was accomplished by maintaining a temperature of 300 K for 500 ps. The NPT (isobaric-isothermal ensemble) was enabled to preserve the persistence of atoms within a complex. In each complex, one pressure bar was maintained with a Berendsen barostat [25]. In addition, MD simulations were conducted for the entire set of complexes by incorporating the SHAKE algorithm to restrain hydrogen bonds [26].

#### 2.3.3. Post-Dynamics MD Analyses

Each enzyme coordinate was saved every 1 ps with its ligand bound. CPPTRAJ integrated into AMBER 18 was used to estimate the trajectory curves in MD simulations [27]. SASA, intramolecular and intermolecular hydrogen bond formation, and thermodynamic calculations of all the systems were calculated. Calculations were conducted on RMSDs of Cα atoms, RMSFs of individual residues in the complex Rg, SASAs, intramolecular and intermolecular hydrogen bond formations, and thermodynamic analyses of all systems. The MD trajectories were analyzed using the Origin tool [28] and the VMD visualization tool [29].

#### 2.3.4. Binding Free Energy Calculations

To calculate all the complexes’ binding free energies, Molecular Mechanics/Generalized Born Surface Area (MM/GBSA) was used [30]. CPPTRAJ was used to remove all counterions and solvents. For each complex, we calculated the binding free energies by using the MM/GBSA method as follows:

Δ*G* = Gcomplex − *Gprotein* − *Gligand* (1)

The free energy term, Δ*Gbind* is calculated using the following equations:

Δ*Gbind* = Δ*Egas* + Δ*Gsolvation* − *T*Δ*S* (2)

where:

Δ*Egas* = Δ*Eint* + Δ*Evdw* + Δ*Eelec* (3)

Δ*Eint* = Δ*Ebond* + Δ*Eangle* + Δ*Etorsion* (4)

Δ*Gsolvation*, = Δ*Gpolar* + *Gnonploar*, (5)

Δ*Gnonploar* = *γ*Δ*SASA* + *β* (6)

When taking binding affinities of ligands in Equation (2), the *T*Δ*S* represents the change in conformational entropy due to ligand binding, which was not considered since it has been found that the entropic contribution when taking binding affinities is often neither reasonable nor necessary. The gas-phase energy (Δ*Egas*) is the sum of internal (Δ*Eint*), van der Waals (Δ*EvdW*), and Coulombic (Δ*Eelec*) energies (Equation (3)). Internal energy (*Eint*) is the amount of energy associated with vibrations of bonds and angles of bonds and rotations of single bonds’ torsional angles (Equation (4)). The solvation free energy (Δ*Gsolvation*) is the sum of polar (Δ*Gpolar*) (*Gnonploar*) and nonpolar (*Gnonploar*) contributions (Equation (5)). Based on the Generalized Born (*GB*) solvation model, the polar solvation contribution was computed using a dielectric constant of 1 for the solute and 80.0 for the solvent [31]. However, the nonpolar free energy (Δ*Gnonploar*) contribution was assessed using Equation (6), where the surface tension proportionality constant, *γ*, and the free energy of nonpolar solvation of a point solute, *β*, were set to 0.00542 kcal mol^−1^ Å^−2^ and 0 kcal mol^−1^, respectively. SASA was calculated via the undeviating arrangement of the pairwise overlap process (LCPO) [32].

### 2.4. In Vitro Methodology

#### 2.4.1. Antifungal Susceptibility

Minimum inhibitory concentration (MIC) of pta-1–pta-3 against three different *C. albicans* isolates was evaluated by broth microdilution assay recommended in the standard M27 document (4th ed.) presented by the Clinical and Laboratory Standards Institute [33]. A fresh colony of *C. albicans* was grown in Sabouraud Dextrose Broth (SDB) at 37 °C for 24 h. After incubation, the culture was centrifuged and resuspended in fresh SDB to achieve a cell suspension of 1.0 × 10^6^ CFU/mL. Initial concentrations of 100 µg/mL of pta1–pta3 were prepared using 1% DMSO (Sigma Aldrich Co., Saint Louis, MO, USA) to test the concentration ranges of 25–0.04 µg/mL. Fluconazole, 1% DMSO, media only, and culture only were used as positive, negative, sterility, and growth controls, respectively. All the plates were incubated at 37 °C for 24 h. The MIC was observed visually as the concentration of piperidine inhibited fungal growth compared to the control.

In addition to the MIC determination, the MFC was determined by subculturing 10 µL from the wells showing no turbidity on SDA (Merck, Johannesburg, South Africa) plates, followed by incubation at 37 °C for 24 h. The lowest concentration with less than 5 colonies on agar plate was recorded as MFC.

#### 2.4.2. Ergosterol Biosynthesis Assay

Further, a range of concentrations (MIC, ½ MIC and ¼ MIC) of pta-1–pta-3 was screened for its effect on ergosterol biosynthesis synthesis in *C. albicans* cells spectrophotometrically, using a method described previously [34]. Briefly, all the three *C. albicans* isolates were treated with ½ MIC and MIC of test compounds, a negative (without test compound), and positive control (8 μg/mL FLC). After 16 h incubation at 37 °C, cells were harvested by spinning at 3000× *g* for 5 min, and the weight of cell pellets was recorded. All the pellets were then dissolved in 3 mL of 25% alcoholic potassium hydroxide solution and vortexed for 1 min. After mixing, cells were incubated for 1 h at 85 °C followed by extraction of sterols by adding 1 ml of sterile distilled water and 3 mL of n-heptane. The cells were then vortexed for 3 min to separate the heptane layer containing total sterols. After 5-fold dilution with 100% ethanol, the heptane layer was scanned spectrophotometrically between 230 and 300 nm. The following equation was used to calculate the ergosterol content as a percentage of the wet weight of the cells:

(7) $$\%ergosterol+\%24\left(28\right)DHE=\frac{\left(\frac{A281.5}{290}\right)\times F}{Pellet\;weight}\;$$

(8) $$\%24\left(28\right)DHE=\frac{\left(\frac{A230}{518}\right)\times F}{Pellet\;weight}\;$$

where *F* = dilution factor, 24(28) DHE = late sterol intermediate, 290 = *E* values for ergosterol, and 518 = *E* values for 24(28) DHE, A281.5 and A230 are the absorbances at 281.5 and 230 nm, respectively.

#### 2.4.3. Gene Expression

The effect of the active derivatives on the *ERG11* gene was studied with RT-qPCR using a previously reported method [35]. All the three tested *C. albicans* strains were exposed to MIC value of piperidine derivatives for 3 h, followed by total RNA extraction and cDNA synthesis using Quick-RNA Fungal Miniprep Kit (Zymo Research, Irvine, CA, USA) and iScriptTM cDNA synthesis kit (Bio-Rad, Hercules, CA, USA) following the manufacturer’s instructions. Purity and concentrations of total RNA were calculated using Nanodrop 2000 spectrophotometer. The relative expressions of *ERG11* gene were determined using PowerUpTM SYBRTM Green Master Mix (2X) (Thermo Fisher Scientific, Waltham, MA, USA). Besides the *ERG11* gene, a housekeeping gene such as *ACT1* was also used as a reference control. Forward and reverse primer sequences for *ERG11* and *ACT1* genes are mentioned in Table S2 and were designed using NCBI/Primer 3-Blast. Differences in amplification were calculated as fold differences and were calculated by using the formula

2 − (*ΔΔCt*) (9)

*ΔCt* = mean *Ct* value of the target gene minus the mean of housekeeping genes; *ΔΔCt* = the *ΔCt* of the tested cells minus *ΔCt* of the control cells.

### 2.5. Effect on Biofilms

#### 2.5.1. XTT Reduction Assay

A commonly used 2,3-Bis(2-methoxy-4-nitro-5-sulfo-phenyl)-2H-tetrazolium-5-carboxanilide (XTT) reduction assay was employed to evaluate the anti-biofilm activity of pta-1, pta-2, and pta-3 against different *C. albicans* isolates. Briefly, *C. albicans* cells at the stationary phase were transferred to different wells of the 96-well plates and were grown for biofilm development at 37 °C for 2 h. After incubation, planktonic cells were removed by washing the wells gently with PBS, followed by the addition of piperidine derivations at different concentrations ranging from 25–0.04 µg/mL. All the plates were incubated for 48 h, and the metabolic activity of adherent cells was calculated by XTT reduction assay by reading the plates at 490 nm using SpectraMax iD3 plate reader (Molecular Devices, San Jose, CA, USA).

#### 2.5.2. Confocal Laser Scanning Microscopy

To study the effect of the active compounds on biofilm formation in different *C. albicans* isolates, a confocal laser scanning microscopy (CLSM) method was used [36], with some modifications. Briefly, *C. albicans* cells were grown under biofilm-forming conditions on cover slips in a 6-well microtiter plate at 37 °C for 24 h. To determine the effect of pta-1, pta-2, and pta-3 on biofilm formation, respective MIC values were added to the designated wells followed by incubation for 2 h. After incubation, planktonic cells were removed, and biofilms were washed gently with PBS. Following washing, biofilms were stained in the dark for 45 min with 10 µM of FUN-1 fluorescent dye (Thermo Fisher Scientific, Johannesburg, South Africa) and concanavalin A (ConA)-Alexa Fluor 488 conjugate (Thermo Fisher Scientific, South Africa). After staining, biofilms were observed using a CLSM-780 and Airyscan (Zeiss 780, Carl Zeiss, Jena, Germany). Multitrack mode was used to collect the images of red (FUN-1) and green (ConA) fluorescence simultaneously. FUN-1 (excitation = 543 nm and emission = 560 nm) is an important dye and is only transported by live cells to reach vacuoles. In dead cells, this dye remains in the cytosol and fluoresces yellow-green, whereas ConA (excitation = 488 nm and emission = 505 nm) binds to α-mannopyranosyl and α-glucopyranosyl residues present in the biofilm matrix and fluoresces bright green color.

### 2.6. Statistics

The data and graphs were made and statistically analyzed using GraphPad Prism 9 (GraphPad Software, San Diego, CA, USA). All the experiments were carried out in triplicates, and the data obtained were presented as means ± standard error of the mean. Two-way ANOVA was used to compare untreated control with treated groups, and a *p*-value ≥ 0.05 was considered significant.

## 3. Results and Discussion

### 3.1. In Silico Results

#### 3.1.1. Molecular Docking Analysis

**Figure 1:**
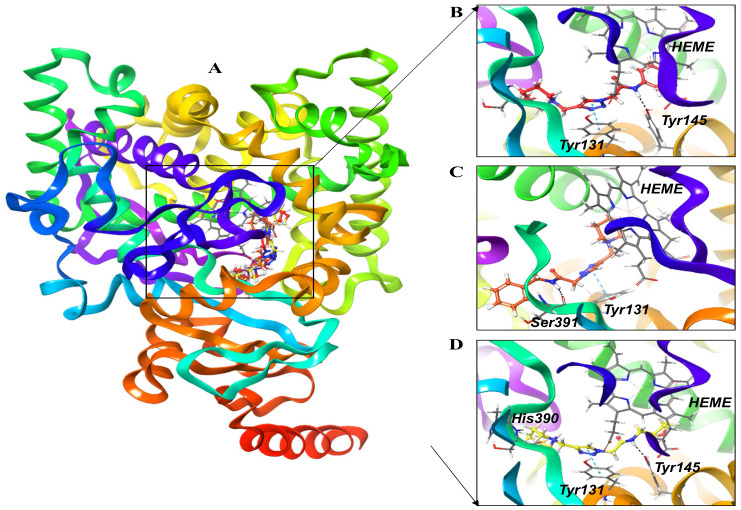
(**A**) Structural representation of LDM enzyme with HEM ion and three superimposed studied inhibitors in the active pocket. Molecular interactions of (**B**) pta1 (red color), (**C**) pta2 (orange color), and (**D**) pta3 (yellow color) with the active site residues of LDM enzyme.

**Figure 2:**
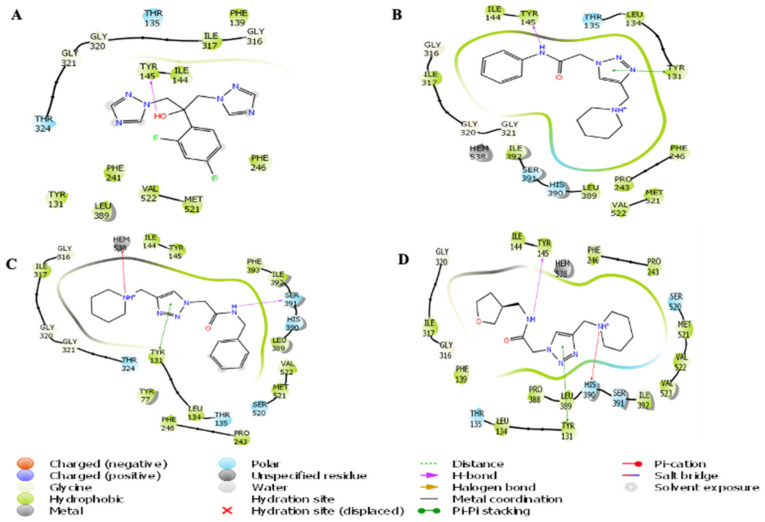
Docked poses of LDM with the studied inhibitors. Molecular interaction of (**A**) Fluconazole and (**B**) pta1, (**C**) pta2, and (**D**) pta3 with the active site of LDM enzyme.

The 3-dimensional structures of the compounds pta1, pta2 pta3, and fluconazole were used to investigate their possible interactions within the active pocket of LDM enzyme by molecular docking approach. We obtained an optimized orientation of the ligands in the active site pocket of an enzyme by minimizing the comprehensive energies of the corresponding complexes. The estimated values of docking scores of all the compounds are shown in Table S1. All three compounds revealed promising binding scores and were thus investigated as lead compounds for LDM inhibition (Figure 1). The pta1-LDM, pta2-LDM, and pta3-LDM complexes showed considerable binding scores with the digital values of −7.19 kcal/mol, −7.53 kcal/mol, and −6.56 kcal/mol (Table S1). Fluconazole-LDM complex revealed the least binding score of −4.80 kcal/mol. The three compounds (pta1, pta2, and pta3) supported LDM interactions through hydrophobic, polar, pi-pi stacking, and metal ion amino acid residues proximate to the binding site of LDM enzyme (Figure 2). The hydroxyl group (–OH) of fluconazole formed one hydrogen bond with Tyr145 (Figure 2A). The amine group (NH) attached to the benzene ring of the pta1 compound produced one hydrogen bond with hydrophobic Tyr145 amino acid residue. One pi-pi interaction of the pta1 compound was also observed with Tyr131 in a complex with LDM enzyme (Figure 2B). The binding score of the pta2 compound was highest compared with other two compounds; it formed one hydrogen bond with Ser391, a pi-pi stacking with Try131, and one pi-cation interaction with metal ion Hem538 (Figure 2C). The molecular docking analysis revealed that pta1, pta2, and pta3 have promising binding scores for inhibiting LDM enzyme over hydrogen-bonding, pi-pi stacking, and pi-cation interactions. Of note, the pta3-LDM complex revealed the least binding score of −6.56 kcal/mol as compared to other two compounds. However, it formed one hydrogen bond with Tyr145, one pi-pi stacking with Tyr131, and a pi-cation interaction with His390 amino acid residue of LDM enzyme (Figure 2D). Therefore, based on the binding score, these three compounds are considered promising binding compounds in complex with LDM enzyme. The binding score of fluconazole was less than the other three compounds. However, all three compounds and fluconazole drug in complex with LDM enzyme were considered for extended structural investigations as fluconazole was used as a positive control in the study against LDM enzyme.

#### 3.1.2. Post-Dynamics Trajectories Analysis

**Figure 3:**
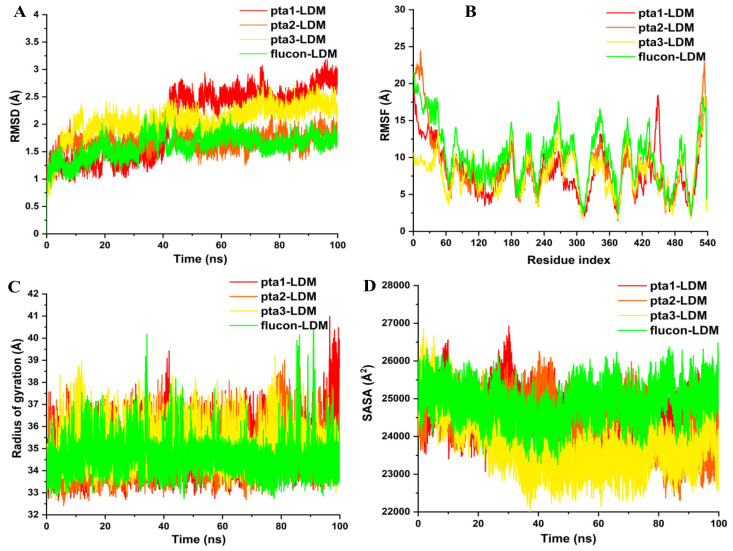
Structural dynamics of LDM enzyme-ligand complexes. (**A**) RMSD, (**B**) RMSF, (**C**) Rg values, and (**D**) SASA values of pta1-LDM, pta2-LDM, pta3-LDM, and fluconazole-LDM in Å across Cα backbone atoms of all the four conditions calculated during the 100 ns of MD trajectories.

The kinetics inside the structure of enzymes are closely related to their genetic behavior. Therefore, the structural integrity of enzymes can be significantly affected by major or minor modifications [37]. The inhibitory action of various enzymes involved in multiple infectious pathways is affected by the binding of small molecules. Hence, there is a requirement to assess the conformational modifications and structural dynamics associated with the inhibitory action of these small molecule compounds [38]. The time-variable estimation regarding RMSD of Cα atoms from generated trajectories was executed to determine the efficiency and consistency of the simulated LDM enzyme in complex with pta1, pta2, pta3, and fluconazole drug. The RMSD perturbational values (Figure 3A) divulged possible symmetrical aberrations in enzyme structure upon binding of studied inhibitors. The plot shown in Figure 3A indicated that all the complexes were steadied and attained convergence after 40 ns of the simulation period. pta2-LDM unveiled the least standard RMSD value of 1.35 Å, followed by fluconazole-LDM with an average value of 1.40 Å, correspondingly. pta1 and pta3-LDM complexes revealed the highest RMSD values of 2.35 Å and 1.97 Å compared to the pta2 LDM complex. This RMSD analysis indicates that pta2-LDM and pta3-LDM complexes showed the least deviation of Cα backbone atoms suggesting that the binding of both the compounds enforced better constancy on the LDM enzyme relative to pta1, and pta3-LDM complexes. The RMSD analysis also proposes that further assessments performed on the produced trajectories for all the complexes were trustworthy.

The stringency and elasticity of the amino acid residues of the enzyme are particularly devoted to the biological activity of the target enzyme in various biotic pathways. Hence, the inhibitor binding to its enzyme might be studied through the alterations in its flexibility regarding the RMSF values [39]. To study the flexibility and rigidity of overall LDM residues upon binding of pta1-pta3 and fluconazole drug, RMSF of Cα atoms were determined from the trajectories throughout 100 ns of MD simulations. As shown in Figure 3B, the pta2-LDM complex indicated the lowest fluctuations in the amino acid residues with an average RMSF value of 7.01 Å. pta1-LDM complex showed a slightly higher RMSF value of 7.46 Å, whereas pta3 and fluconazole LDM complexes exhibited higher RMSF values with 9.05 Å and 9.85 Å in comparison with other LDM complexes, indicative of a convincing activity of pta2 and pta1 against LDM enzyme. The influential binding of these two compounds at the catalytic site might be correlated with the structural inactivation of LDM enzyme.

Additionally, to substantiate our results, we have determined the Rg values, which is a factor correlated with the aggregate conformational modifications within enzyme structure after inhibitor binding. It also uncovers enzymes structural compactness, folding behavior, and its stability [40]. The compactness of pta1-LDM, pta2-LDM, pta3-LDM, and fluconazole-LDM complexes was determined by estimating their Rg values. pta2-LDM complex revealed a lowest Rg value of 34.12 Å, whereas a slight increase was noted in the Rg value of 34.25 Å of the fluconazole-LDM complex, as shown in Figure 3C. pta1 and pta3 LDM complexes exhibited higher Rg values of 35.21 Å and 35.33 Å in comparison with the other two studied compounds. This analysis indicated improved compactness and enhanced activity of pta2 upon LDM binding. All these conformational dynamics analyses signify enhanced steadiness, elasticity, and compactness of the three tested compounds with LDM enzyme.

After analyzing the conformational binding, SASA was measured to define the utility of hydrophobic and hydrophilic amino acid residues and energies shown to the solvent throughout the simulation time [40]. The rapid and accurate estimation of SASA is highly advantageous in the energetic assessment of biotic compounds. The connections among the hydrophobic intrinsic contacts inside enzyme structure is a special intermolecular connection that affects enzyme inhibition. These hydrophobic connections generated between the non-polar amino acid residues corroborate the steadiness of enzyme structure inside solution by guarding the non-polar residues in the hydrophobic core distant from an aqueous mixture [19]. Overall SASA values for pta1-LDM, pta2-LDM, pta3-LDM, and fluconazole-LDM complexes were measured throughout the 100 ns MD simulation production run (Figure 3D). The standard SASA value of 23,015 Å2 was noted in pta3-LDM complex after the complex was exposed to the solvent. A slight increase in an average SASA value of pta2-LDM complex was observed at 23,503 Å2 compared to pta3 and fluconazole LDM enzyme, which was noted at 27,023 Å2 and Å2 27,224, respectively. The pta1-LDM complex exposed a higher SASA value against other three LDM complexes; however, it does not indicate the least binding of pta1 compound as other analysis supports the enhanced activity of this compound. The alterations in SASA values for the four complexes throughout the simulation period feature the folding and unfolding behavior of LDM enzyme. A negligible change in overall SASA values of pta1–pta3 compounds with LDM complexes was observed. Therefore, the results confirm that the enhanced exposure to solvent led to the increased inhibitory activity of pta1, pta2, and pta3 over the fluconazole LDM enzyme complex.

#### 3.1.3. Hydrogen Bond Analysis

**Figure 4:**
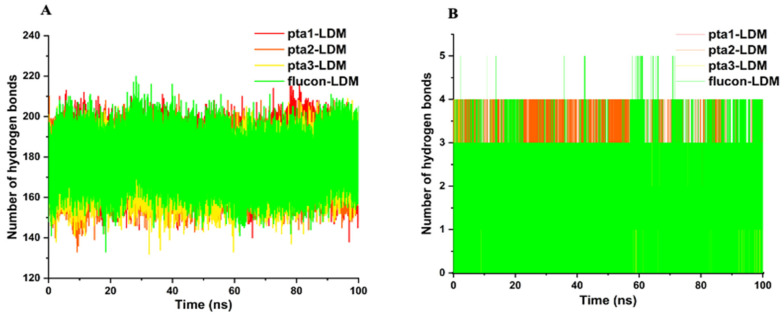
Hydrogen bond analysis. (**A**) Intramolecular hydrogen bonds in pta1, pta2, pta3, and fluconazole-LDM complexes. (**B**) Intermolecular hydrogen bonds in pta1, pta2, pta3, and fluconazole bound LMD complexes calculated after 100 ns MD simulation.

Intramolecular and intermolecular hydrogen bond analysis plays an important role in assessing the overall conformation and stability of enzyme structure (Figure 4). The evaluation offers an extensive understanding into the enzyme-ligand binding mechanism with detailed observation. A standard value of intramolecular hydrogen bonds in the pta2-LDM complex was noted between 170–175, whereas it was 160–176 in the pta3-LDM complex correspondingly, as shown in Figure 4A. The average number of hydrogen bonds in pta1 and fluconazole complexes were observed between 175–180 and 176–182, respectively (Figure 4A). A notable characteristic of the fluconazole-LDM complex is the number of hydrogen bonds produced around the active pocket of the LDM enzyme. However, pta1, pta2, and pta3-LDM complexes only had 1–2, 2–3 hydrogen bonds where fluctuations were lowest (Figure 4B).

#### 3.1.4. Mechanistic Insight into Free Binding Energy

The impact of the ligand’s binding free energy on the total binding free energy of the complex is related to the structural stability of the ligand in the active pocket of its target enzyme. The intermolecular hydrogen bond, pi-pi interactions, and other valuable interactions produced between the active or allosteric site amino acid residues contribute significantly to the binding affinity, selectivity, and stability of the ligand. Hence, it was essential to measure the binding affinity of pta1–pta3 and fluconazole drug in complex with LDM enzyme using an MM/GBSA approach to determine the effect of three compounds on LDM enzyme. The results obtained are shown in Table 1.

A predictable free binding energy (Δ*Gbind*) of pta1-LDM complex was measured as the highest energy with a standard digital value of −43.66 kcal/mol compared to pta2-LDM and pta3-LDM complexes with average values of −40.10 kcal/mol and −42.66 kcal/mol. Fluconazole-LDM complex reveals the lowest binding energy value of −31.27 kcal/mol. To validate the overall binding free energy values, other components of the free binding energy correlated with ligand-enzyme binding were also assessed.

It has been noted that the intermolecular van der Waals and electrostatic energies in the pta1-LDM complex were more promising, with standard values of −41.52 and −19.39 kcal/mol. However, the van der Waals energy is slightly higher with the digital value of −41.98 kcal/mol in pta2-LDM complex. The electrostatic energy of pta2-LDM complex was lower (−18.17 kcal/mol), whereas it was higher in pta3-LDM complex with a value of −21.64. The gas-phase energy (Δ*Ggas*) was somewhat similar in pta2 (−61.67 kcal/mol) and pta3-LDM (−61.45 kcal/mol) complexes. However, gas-phase energy (Δ*Ggas*) was highest in the pta1-LDM complex with a value of −67.92 kcal/mol. The solvation energy (Δ*Gsol*) was also slightly higher with the value of 36.45 kcal/mol in pta1-LDM complex, whereas the same energy has been noted to be 32.76 kcal/mol and 26.18 kcal/mol in pta2 and pta3-LDM complexes. This analysis provides an understanding about the considerable binding of pta1, pta2, and pta3 compounds to LDM enzyme with increased affinity.

### 3.2. In Vitro Results

#### 3.2.1. Antifungal Susceptibility 

The antifungal susceptibility of piperidine derivatives (pta1–pta3) was tested by determining their MICs. The MIC values for these derivatives ranged from 0.024–3.125 µg/mL (Table 2), with pta1 having the lowest MIC value and pta3 with the highest MIC value. As expected, the most susceptible strain with the lowest MIC values was the laboratory strain *C. albicans* SC5314 followed by *C. albicans* 4175 (fluconazole susceptible) and *C. albicans* 5112 (fluconazole resistant) with highest MIC values ranging from 0.390–3.125 µg/mL.

MFC of pta1–pta3 was also determined and, similar to MIC values, pta1 had the lowest MFC of 0.097 and pta3 had the highest MFC value of 6.250 (Table 2). MFC values were one- to two-fold higher than MIC values, except that of pta2 against *C. albicans* 5112, where both MIC and MFC values were the same.

#### 3.2.2. Ergosterol Biosynthesis 

**Figure 5:**
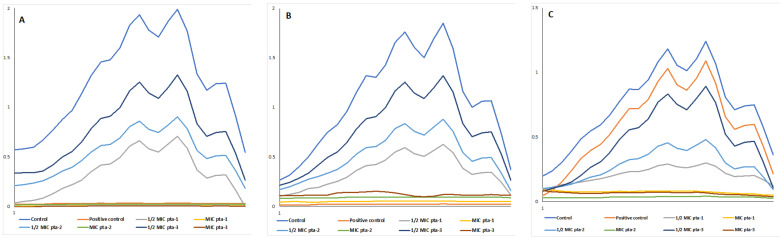
Effect of piperidine based derivatives (pta-1–pta-3) on ergosterol levels in *C. albicans* SC5314 (**A**), *C. albicans* 4175 (**B**) and *C. albicans* 5112 (**C**). Positive controls are represented by the cells treated with 8 μg/mL of fluconazole while negative controls are represented by healthy cells.

Based on in silico results displaying the interaction of piperidine-based derivatives (pta–pta3) with LDM, the effect of these derivatives on ergosterol biosynthesis in *C. albicans* isolates was studied. Figure 5 represents the 4-peak graphical presentation of ergosterol biosynthesis and its inhibition by piperidine derivatives at different tested concentrations in *C. albicans* SC5314 (Figure 5A), *C. albicans* 4175 (Figure 5B) and *C. albicans* 5112 (Figure 5C).

To further quantify the effect of pta-1–pta-3 on ergosterol biosynthesis, %-reduction in ergosterol content was calculated and represented in Table 3. A dose-dependent decrease in the ergosterol biosynthesis was observed when cells were treated with piperidine compounds. The %-age decrease in ergosterol content at ½ MIC and MIC values of pta1 was higher compared to pta2 and pta3. In *C. albicans* SC5314, the total reduction in ergosterol biosynthesis ranged from 33 to 64% at ½ MIC and 91 to 96% at MIC values of piperidine-based derivatives. For *C. albicans* 4175, these values range from 28–66% and 93–97% at ½ MIC and MIC values of the test compounds, respectively. Similarly, for *C. albicans* 5112 at ½ MIC and MIC values of piperidine-based derivatives, the %-age decrease of ergosterol synthesis was lowered in the range of 23–74% and 91–96%, respectively. 

Fluconazole (8 μg/mL) in *C. albicans* SC5314 and *C. albicans* 4175 decreased the ergosterol production by >95%, whereas in fluconazole-resistant strain, *C. albicans* 5112, only 13% decrease was recorded.

The above results are congruent with the in silico findings, where LDM–pta1, LDM–pta2, and LDM–pta3 complexes altered the protein conformation, with LDM–pta1 being the most active among the three. Furthermore, the high negative binding energies of interaction by pta1 compared to pta2 and pat3, indicative of a more promising binding affinity, could account for the favorable ligand binding. Despite significant differences, all three derivatives showed promising interaction with LDM enzyme, making a way to test their in vitro effect on the ergosterol biosynthesis pathway. In both susceptible and resistant *Candida* isolates, the reduction of ergosterol in a dose-dependent manner has been reported previously with natural and synthetic compounds. Our results in this study further confirm the dose-dependent inhibition of ergosterol biosynthesis [12,34,35]. The ergosterol biosynthesis pathway has traditionally been the target of antifungal drugs, including azoles and polyenes. In addition to these two established antifungal drug classes, this pathway and the enzymes involved have been extensively studied as a potential drug target. Dose-dependent decrease of ergosterol biosynthesis by these derivatives is in line with the previous findings, where natural and synthetic compounds were reported to inhibit the ergosterol biosynthesis pathway [41,42]. Due to the evolutionary relationship between human hosts and the fungal pathogens, finding unique differences between human and fungal cells is a great challenge. As a result of the difference between these eukaryotic cells, ergosterol has become a focus for drug development concerning antifungal therapy. Furthermore, ergosterol is an important component of fungal cell membranes and has been well studied owing to its importance in maintaining the physiology, integrity, fluidity, and rigidity of *Candida* cells [43]. In addition, ergosterol has also been reported to maintain membrane heterogeneity coordination and prevent water penetration in fungal cells [43]. All these factors highlight the importance of ergosterol in the survival of this pathogen; therefore, depletion of this sterol has been utilized for drug development. Additionally, these results provide strong evidence that triazole derivatives can deplete ergosterol synthesis and inhibit growth, suggesting their potential use as antifungal drugs.

#### 3.2.3. Gene Expression

**Figure 6:**
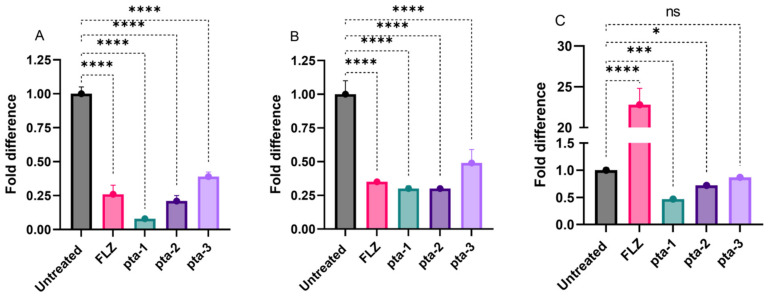
Quantification of *ERG11* gene expression after normalization to *ACT1* in different *C. albicans* strains after exposed to inhibitory concentrations of pta-1, pta-2, and pta-3. (**A**). *C. albicans* SC5314. (**B**) *C. albicans* 4175. (**C**) *C. albicans* 5112. Results are the mean ± SD of three different readings. Bars with asterisks indicate significant differences as follows (*** *p* < 0.001). **** *p* < 0.0001; *** *p* = 0.002; * *p* = 0.0171; ns indicates non-significant = 0.3976.

To further test the ability of these derivatives (pta1–pta3) to inhibit *ERG11* gene expression in different *C. albicans* isolates, cells were exposed to MIC values, and the results are summarized in Figure 6. In comparison to untreated cells, a significant decrease in the gene expression was observed when cells were exposed to the pta1 followed by pta2 and pta3. Piperidine compounds pta1, pta2, and pta3 at their respective inhibitory concentrations have been recorded to downregulate the expression of *ERG11* by 2.2–4.8-folds, 0.7–3.8-folds, and 0.3–2.2-folds, respectively, compared to untreated control cells that were set to 1.0 (Figure 6). These results reinforce that the triazole compounds downregulated the expression of *ERG11*, which in turn led to the depletion of 4,4-dimethylcholesta trienol and thereby inhibited ergosterol biosynthesis pathway in *C. albicans*.

The ergosterol biosynthesis pathway is a well-reported target for azoles, allylamines, and morpholines, which inhibit lanosterol 14-α demethylase (*ERG11*), squalene epoxidase (ERG1), and sterol C-14 reductase (ERG24)/sterol C-8 isomerase (ERG2) [28,29,30]. Thus, the enzymes and their respective genes in the ergosterol biosynthesis pathway serve as antifungal drug targets. Mutations in *ERG11* are directly linked to azole drug resistance in *C. albicans* [44,45]. Disruption of ergosterol biosynthesis pathway by inhibiting *ERG11* results in the increase of lanosterol, further damaging the cell membrane and integrity [33]. On the contrary, the overproduction of *ERG11* protein results in azole and polyene resistance in *Candida* isolates [46]. In this study, we observed various levels of downregulation of the *ERG11* gene in each of the tested triazole derivatives. The impact of the downregulation of this gene is evident from the ergosterol quantification results. Previously, several natural and synthetic compounds have been reported to decrease the expression of the *ERG11* gene with varying effects on ergosterol production [33,34,47].

#### 3.2.4. Biofilm Formation

*Candida* biofilm mass, growth, architecture, and potential of piperidine derivatives to inhibit biofilm formation were studied quantitatively by XTT reduction assay and qualitatively by viewing the biofilms under Confocal laser scanning microscopy (CLSM). XTT is the most common method used to study the *Candida* biofilm mass and response to drug therapies. The results clearly showed the antibiofilm activity of the test piperidine derivatives against all the different *C. albicans* isolates at varying levels. Biofilm MICs were determined as the lowest concentrations which inhibit ≥80% metabolic activities in comparison to untreated healthy control cells which could form dense biofilm on the coverslip. At planktonic MIC values, all the test derivatives inhibit the biofilm formation by ≥50%, whereas the biofilm MIC was recorded as 1-fold higher than the planktonic MIC values.

**Figure 7:**
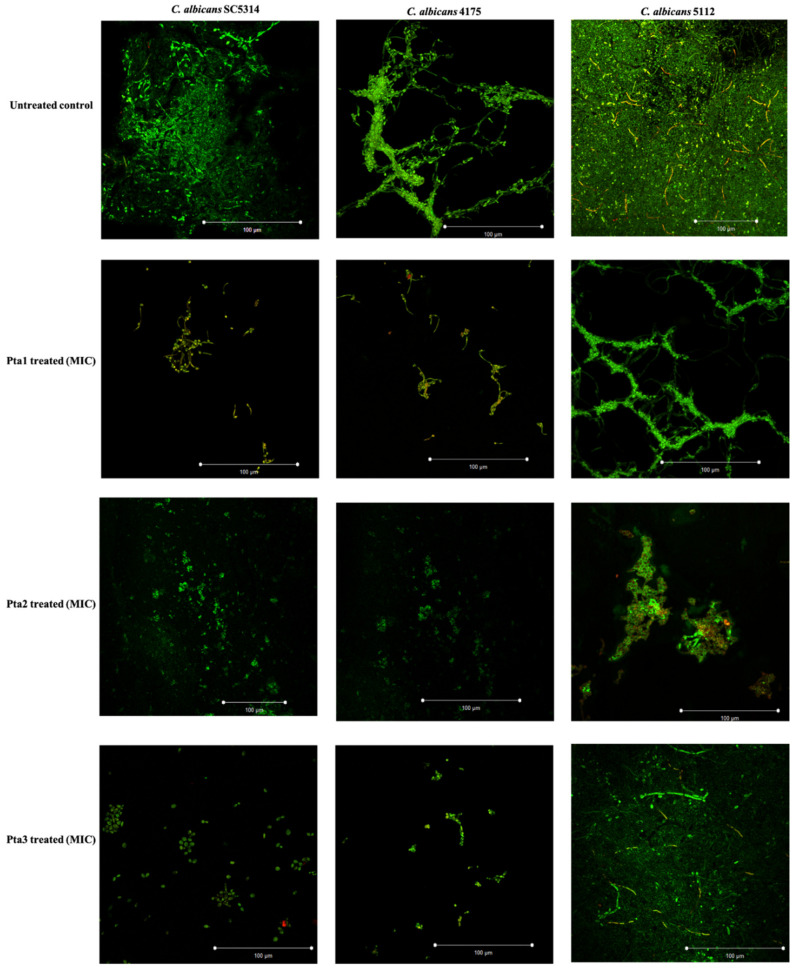
Anti-biofilm activity of piperidine derivatives. Representative figure of different *C. albicans* biofilms after exposure to pta-1, pta-2, and pta-3 at their inhibitory concentrations. The green fluoresces (Con A) represent the *C. albicans* cell wall as well as biofilm matrix; yellow-green fluoresces (FUN-1) represent dead/metabolically inactive cells.

To further confirm the antibiofilm activity of the piperidine derivatives, CLSM was undertaken. The high number of aggregated and metabolically inactive cells in untreated slides are visible by the presence of yellow-green fluorescence (Figure 7). The biofilms in control untreated slides were well-defined and dense biofilm matrix with bright green fluorescence. The results also marked that the biofilms of fluconazole resistant *C. albicans* 5112 were atypical 3-D structure, composed of hyphae. From Figure 7, it is evident that MIC values of pta-1, pta-2, and pta-3 abrogate the viability and biofilm formation in all the tested *C. albicans* isolates to varying extents. Furthermore, in presence of the piperidine compounds, the biofilm lacks true hyphae and mostly comprises pseudohyphae and yeast cells only. These results clearly indicate that the piperidine derivatives affected the sustainability of yeast cells in biofilms and thereby confirm the ability of these piperidine derivatives to penetrate *Candida* cell envelops resulting in effective anti-biofilm activity.

## 4. Conclusions

This study reported the antifungal activity of triazole-based derivatives (pta1, pta2, and pta3) against fluconazole-susceptible and -resistant *C. albicans* strains. In-depth in silico and in vitro studies revealed that these compounds have the potential to interact with the LDM enzyme and downregulate the expression of *ERG11* gene, which in turn inhibits the ergosterol biosynthesis pathway. This study builds on previous reports that these compounds induce apoptosis and cell cycle arrest, suggesting the potential to be developed as novel antifungal drugs with multiple mechanisms of action, thereby minimizing drug resistance. Furthermore, the low cytotoxicity of these compounds has already approved the use of these compounds for in vivo studies. To validate these claims, however, further research is needed to understand their antifungal mechanisms and test these derivatives against different *Candida* species in animal models.

## Figures and Tables

**Table 1:** MM/GBSA-based binding energy profile of LDM enzyme in complex with pta1, pta2, pta3, and fluconazole (FLZ).

Complex	Δ*E*_vdW_	Δ*E*_ele_	Δ*G*_gas_	Δ*G*_polar_	Δ*G*_nonpolar_	Δ*G*_sol_	Δ*G*_bind_
pta1-LDM	−41.52	−19.39	−67.92	41.03	−4.58	36.45	−43.66
pta2-LDM	−41.98	−18.17	−61.67	39.07	−6.31	−32.76	−40.10
pta3-LDM	−39.80	−21.64	−61.45	30.43	−4.26	26.18	−42.66
FLZ-LDM	−43.98	−14.32	−58.37	32.21	−5.16	27.04	−31.27

**Table 2:** MIC and MFC values of piperidine based derivatives against *C. albicans* isolates.

Isolates	Fluconazole Susceptibility	pta1 (µg/mL)		pta2 (µg/mL)		pta3 (µg/mL)		FLZ
		MIC	MFC	MIC	MFC	MIC	MFC	
*C. albicans* SC5314	Susceptible	0.024	0.097	0.097	0.390	0.781	1.560	0.250
*C. albicans* 4175	Susceptible	0.048	0.097	0.097	0.390	1.560	3.125	0.125
*C. albicans* 5112	Resistant	0.390	0.781	1.560	1.560	3.125	6.250	64.00

**Table 3:** Percentage reduction of ergosterol biosynthesis.

Candida Strains	Test Compounds	Mean Ergosterol Content *	
*C. albicans* SC5314	Negative control	0.0231	
	Positive control	0.00027 (99) **	
	pta1	½ MIC	0.00834 (64) **
		MIC	0.00085 (96) **
	pta2	½ MIC	0.01035 (55) **
		MIC	0.00131(94) **
	pta3	½ MIC	0.01543 (33) **
		MIC	0.00208 (91) **
*C. albicans* 4175	Negative control	0.01911	
	Positive control	0.00037 (98) **	
	pta1	½ MIC	0.00647 (66) **
		MIC	0.00055 (97) **
	pta2	½ MIC	0.00915 (52) **
		MIC	0.00093 (95) **
	pta3	½ MIC	0.0137 (28) **
		MIC	0.0013 (93) **
*C. albicans* 5112	Negative control	0.01311	
	Positive control	0.01141 (13) **	
	pta1	½ MIC	0.00341 (74) **
		MIC	0.00054 (96) **
	pta2	½ MIC	0.0055 (58) **
		MIC	0.00089 (93) **
	pta3	½ MIC	0.01007 (23) **
		MIC	0.0012 (91) **

* Calculated as % wet weight of the cells (% reduction in the ergosterol content compared to controls). ** Significant reduction (*p* < 0.05).

## Data Availability

The data available are provided in this manuscript.

## Supplementary Materials

The following supporting information can be downloaded at: https://www.mdpi.com/article/10.3390/jof8070688/s1, Scheme S1: Synthesis of piperidine based 1,2,3-triazolylacetamide derivatives (pta1-pta3); Table S1: Docking profile of pta1, pta2, pta3 and fluconazole in complex with LDM enzyme; Table S2: List of primers for ergosterol synthesis gene for RT-qPCR.
