# Supplementary material for: Triazole Derivatives Target 14α–Demethylase (LDM) Enzyme in Candida albicans Causing Ergosterol Biosynthesis Inhibition
Source: J Fungi (Basel). 2022 Jun 29;8(7):688. doi: 10.3390/jof8070688 (PMC9323696; doi:10.3390/jof8070688)
Supplement: Supplementary file 1 [file jof-08-00688-s001.zip › jof-1714292-supplementary resubmitted.pdf]

Supporting Information

Candida albicans causing ergosterol biosynthesis inhibition

Irfan A. Rather [1,2,*], Jamal S.M. Sabir[1,2], Amer H. Asseri[3], Mohmmad Younus Wani[4,*], and Aijaz Ahmad[5,6,*]

[1] Department of Biological Sciences, Faculty of Science, King Abdulaziz University (KAU); Jeddah 21589, Saudi Arabia; jsabir@kau.edu.sa
[2] Centre of Excellence in Bionanoscience Research, King Abdulaziz University (KAU), Jeddah 21589, Saudi Arabia
[3] Biochemistry Department, Faculty of Science, King Abdulaziz University (KAU), Jeddah 21589, Saudi Arabia; ahasseri@kau.edu.sa
[4] Department of Chemistry, College of Science, University of Jeddah, P.O. Box 80327, Jeddah 21589, Saudi Arabia; mwani@uj.edu.sa
[5] Clinical Microbiology and Infectious Diseases, Faculty of Health Sciences, School of Pathology, University of Witwatersand, Johannesburg 2193, South Africa
[6] Infection Control Unit, Charlotte Maxeke, Johannesburg Academic Hospital, National Health Laboratory Service, Johannesburg 2193, South Africa; aijaz.ahmad@witz.ac.za
* Correspondence: ammm@kau.edu.sa (IAR); aijaz.ahmad@witz.ac.za (AA); mwani@uj.edu.sa (YW))

Table S1: Docking profile of pta1, pta2, pta3 and fluconazole in complex with LDM enzyme.

Table S2: List of primers for ergosterol synthesis gene for RT-qPCR

Scheme S1: Synthesis of piperidine based 1,2,3-triazolylacetamide derivatives (pta1-pta3)

Table S1: Docking profile of pta1, pta2, pta3 and fluconazole in complex with LDM enzyme.

Compounds	Docking Score (kcal/mol)
**pta-1**	-7.19
**pta-2**	-7.53
**pta-3**	-6.56
**Fluconazole**	-4.80

Table S2: List of primers for ergosterol synthesis gene for RT-qPCR

Gene	Primer	Sequence (5′–3′)	Amplicon length (bp)
ERG11	Forward	*CTC ATG GGG TTG CCA ATG TT TTT*	207
	Reverse	*GAG CAG CAT CAC GTC TC*	
ACT1	Forward	*TGG TGA TGA AGC CCA ATC CA CAT*	169
	Reverse	*TGG AGC TTC GGT CAA CA*	

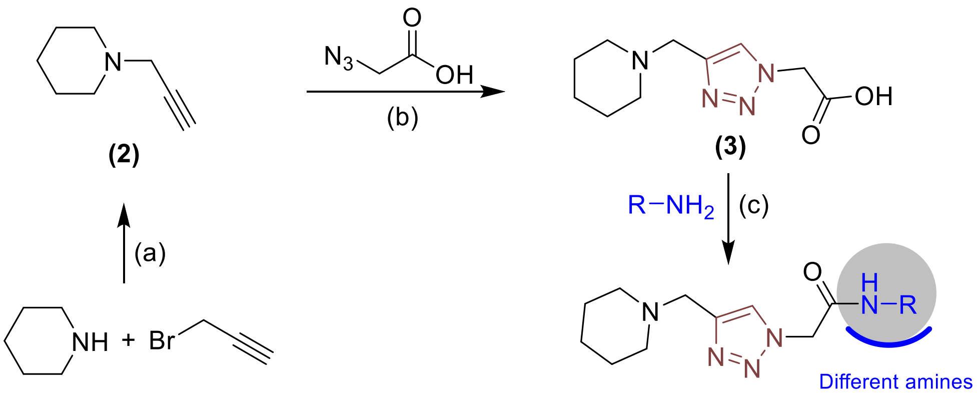

pta1-pta6

Reagents and conditions: (a) Dry Acetone, r.t, 1 min (b) Sodium ascorbate, $CuSO_4.5H_2O$, DMF (c) HATU, DIPEA, DMF, 50 °C.

**Compound**	**R-NH$_2$** **Different amines**
**pta1**	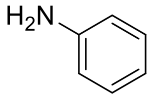
**pta2**	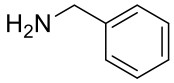
**pta3**	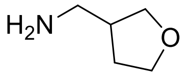

Scheme S1: Synthesis of piperidine based 1,2,3-triazolylacetamide derivatives (pta1-pta3)
